# ﻿A new species of the genus *Oiketicoides* Heylaerts, 1885 (Lepidoptera, Psychidae) from Korea with its natural parasitoid enemy

**DOI:** 10.3897/zookeys.1223.135001

**Published:** 2025-01-14

**Authors:** Dong-June Lee, Jongwon Kim, Seung-Su Euo, Jae-Seok Lee, Hyeon Lee, Seung Jin Roh

**Affiliations:** 1 Division of Zoology, Honam National Institute of Biological Resources, Mokpo, Republic of Korea Honam National Institute of Biological Resources Mokpo Republic of Korea; 2 Division of Forest Biodiversity, Korea National Arboretum, Pocheon 11186, Republic of Korea Korea National Arboretum Pocheon Republic of Korea

**Keywords:** Bagmoths, bagworms, DNA barcode, Psychidae, taxonomy

## Abstract

*Oiketicoidesgohadoensis* Roh & Lee, **sp. nov.** is described as new to science. The morphology of male adult, including genitalia, is described, and DNA barcodes for precise identification of the species are provided. A parasitoid, *Neophryxepsychidis* Townsend, 1916 (Diptera, Tachinidae) of *O.gohadoensis* is also reported for the first time in Korea, together with its DNA barcode sequence.

## ﻿Introduction

The family Psychidae, so-called bagworms or bagmoths, consists of 241 genera with 1350 described species (van Nieukerken et al. 2011). Phylogenetically, Psychidae have been placed in the superfamily Tineoidea (Regier et al. 2015).

The genus *Oiketicoides* Heylaerts, 1885 is based on the type species *Psyche inquinata* Lederer, 1858, by subsequent designation by Hampson in 1892 (Sobczyk 2011). In total, 45 species of *Oiketicoides* are known, with 40 species distributed throughout the Palaearctic Region (Arnscheid and Sobczyk 2023). In East Asia, only one species, *Oiketicoidesorophila* (Wehrli, 1928) is known from Xinjiang Province, China (Jia and Wu 2023). *Oiketicoides* species occur in dry habitats at high altitudes up to 3000 m. Almost all species of *Oiketicoides* are uniformly dark yellowish-brown or grey in colour and characterized by the following adult morphological characters: the decreasing length of the antennal pecten towards the antennal tip; a very long tibial epiphysis on the foreleg; and 11 veins on the forewing and seven veins on the hindwing (Arnscheid and Sobczyk 2023). The larvae live mostly hidden on the ground without showing any preference for particular food plants (Arnscheid and Sobczyk 2023).

In this study, *Oiketicoidesgohadoensis* sp. nov. is described as new. All available information is presented, including the collection locations, microhabitats, and illustrations of male and its genitalia. DNA barcodes are provided for precise identification. A parasitoid dipteran, *Neophryxepsychidis* Townsend, 1916 (Tachinidae) of *O.gohadoensis* was reported for the first time in Korea.

## ﻿Material and methods

The material examined in this study is kept in the Insect Collection, Honam National Institute of Biological Resources (**HNIBRIN**), Mokpo, Korea. The male genitalia were dissected and examined after mounting on glass slides in 80% glycerol solution. The wing venations were examined in 70% alcohol solution. Photographs of adults were taken using a Canon MP-E 65 mm f/2.8 1–5 × macro lens attached to Canon 5D Mark IV digital camera (Canon, Tokyo, Japan). Photographs of the male genitalia were taken using a DFC 95 mm digital camera (Leica, Wetzlar, Germany) attached to a Leica M205A stereomicroscope (Leica, Wetzlar, Germany). Terminology and morphological characters of the adult, wing venation and genitalia follow Saigusa and Sugimoto (2014) and Arnscheid and Weidlich (2017).

Genomic DNA from three specimens of *Oiketicoidesgohadoensis* sp. nov. and one specimen of *Neophryxepsychidis* was extracted from the legs of dried specimens of adults in 100% alcohol using a DNeasy Blood and Tissue kits (Qiagen, Inc, Hilden, Germany) according to the manufacturer’s protocol. Specimens were sequenced and the DNA barcode, cytochrome *c* oxidase subunit I gene (COI), was amplified using the primers LCO1490 and HCO2198 (Folmer et al. 1994). Polymerase Chain Reaction (PCR) conditions for amplification followed the manufacturer’s protocol (Platinum Taq, Invitrogen, Carlsbad City, CA, USA). The amplicons were purified using the QIAquick® PCR purification kit (QIAGEN, Inc, Hilden, Germany) and directly sequenced at Macrogen (Seoul, Korea). Contigs were assembled in Geneious Prime (Kearse et al. 2012). Successful sequences were uploaded to GenBank (*O.gohadoensis*: PP983255–PP983257 and *N.psychidis*: PP983258).

## ﻿Results

### ﻿ Oiketicoides

Taxon classificationAnimaliaLepidopteraPsychidae

Heylaerts, 1881

D30C18F3-6333-56DA-B7F3-620D23CC4C75

Acanthopsyche (Oiketicoides) Heylaerts, 1881. *Annales de la Société entomologique de Belgique* 25: 66.

#### Type species.

*Psyche inquinata* Lederer, 1858. Wiener entomologische Monatschrift 2(5): 142.

### ﻿ Oiketicoides gohadoensis

Taxon classificationAnimaliaLepidopteraPsychidae

Roh & Lee sp. nov.

DD4509ED-3F6C-58B1-9E9A-DAA5CB7D75CF

https://zoobank.org/B284172B-2DE9-4138-B308-A07E96EEB422

#### Type material.

***Holotype***: ♂, South Korea • Mokpo, Gohado Island; 28.vi.2023; 34°46'01"N, 126°22'02"E; altitude 12 m; leg. J.W. Kim; HNIBRIN 16107.

***Paratypes***: 9♂; same label data as holotype; HNIBRIN 16104–16106, 16108–16113.

#### Diagnosis.

The genus *Oiketicoides* has little difference in external morphological characters between the males of the species, making morphological diagnosis difficult (Arnscheid and Sobczyk 2023). This new species also appears to have typical characters (uniformly dark, yellowish-brown coloration), but it has a noticeably shorter wingspan (11–13 mm) compared to the other species. The male genitalia of *O.gohadoensis* are very similar to those of *O.elegantis* Arnscheid & Sobczyk, 2023, but the cucullus is wider and club-shaped. Moreover, the male genitalia differ in having a wide vinculum and a downwardly thick saccus.

#### Description.

**Figure 1.**
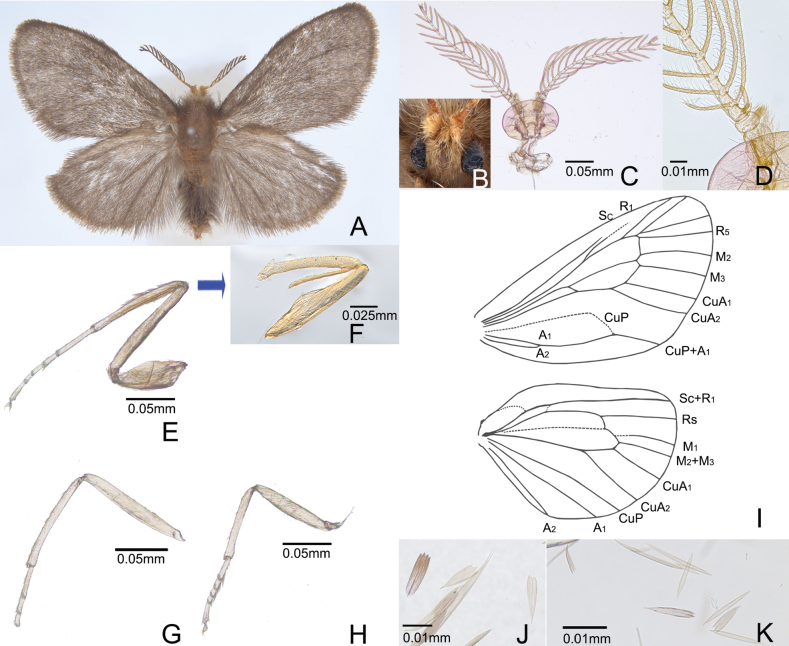
Adult *Oiketicoidesgohadoensis* Roh & Lee, sp. nov. **A** male of adult **B** anterior view of head **C** anterior view of head and antennae **D** basic view of antenna **E** foreleg **F** tibial epiphysis of foreleg **G** midleg **H** hindleg **I** wing venation **J** forewing scales **K** hindwing scales.

***Adult*** (Fig. 1). Male. Head: vertex densely clothed with yellowish-brown hairs; ocelli absent; antennae less than length of 2/5 of forewing, scape roughly covered with hairs, bipectinate, with 14 flagellomeres. Thorax: notum covered with dark, yellowish-brown scales. Legs with femora, tibiae, and tarsi clothed in light-brown hairs; tarsi and apical and medial spurs covered by yellowish-brown scales; foreleg with a long and narrow tibial epiphysis. Wingspan 11–13 mm. Forewing dark brown, mostly covered with short, hair-like scales; accessory and intercalary cells absent; 10 separate veins originating at discal cell; Sc terminating at 3/5 of costa; R3 and R4 stalked at anterior part of cell to reach apex; M2 and M3 parallel; scales slightly narrowed; apical margin usually produced into 2–4 weak, rounded lacininations. Hindwing covered with dark-brown scales; 6 veins from discal cell; M2 + M3 fused; scales narrowed; apical margin usually produced into two or three weak, rounded lacininations.

**Figure 2.**
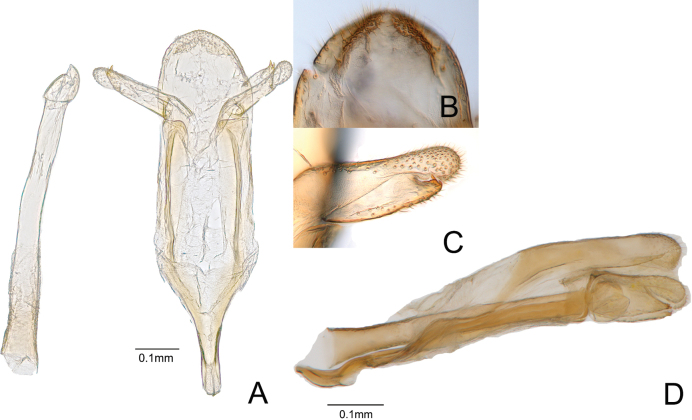
Male genitalia of *Oiketicoidesgohadoensis* Roh & Lee, sp. nov. **A** dorso-ventral aspect **B** tegumen **C** valva **D** lateral aspect.

***Male genitalia*** (Fig. 2). Tegumen wide, slightly folded; valva short and slender; sacculus sclerotized with short setae; cucullus arched, club-shaped; vinculum slightly narrow; saccus straight, slightly thick, and long; phallus thick and long, 0.87 times as long as genitalia.

#### Distribution.

Korea (new species).

#### DNA barcode.

DNA barcode sequences were generated from three specimens of *Oiketicoidesgohadoensis* sp. nov. (PP983255, PP983256, and PP983257). Multiple alignments using the BLAST tool in the NCBI database showed *Claniaignobilis* Grote, 1873 to be the nearest neighbor at 86.49%. The maximum intraspecific genetic variation ranged from 1.09 to 0.62%, a little lower than interspecific distances.

#### Etymology.

**Figure 3.**
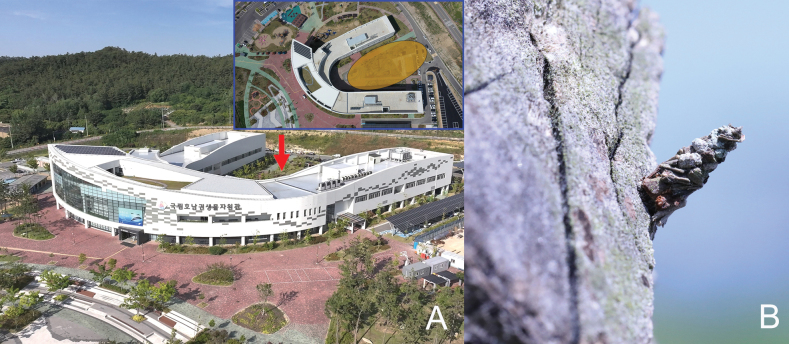
Microhabitat and larval case of *Oiketicoidesgohadoensis* Roh & Lee, sp. nov. **A** microhabitat in my office garden (Korea: Honam National Institute of Biological Resource, Gohado Island, Mokpo-si, Jeollanam-do, 1.vi.2023, 34°.46'01"N, 126°22'02"E, altitude 12 m) **B** larval case.

This species was discovered on a tree in the garden at Honam National Institute of Biological Resources, Mokpo, Korea (Fig. 3A). The specific name is derived from the type locality (Gohado Island) of the new species.

#### Biology.

Larvae of the new species build their cases (8.1–10.3 mm in length) by adhering the tiny particles of bark to their case. In addition, they were found to live in dried conditions between the bark or leaves of trees (Fig. 3). Adults emerge from late June to mid-July in breeding condition. Of 11 larvae, 11 males emerged in the present study.

##### ﻿Natural enemy of *O.gohadoensis*

###### ﻿*Neophryxepsychidis* Townsend, 1916 (Diptera, Tachinidae)

**Figure 4.**
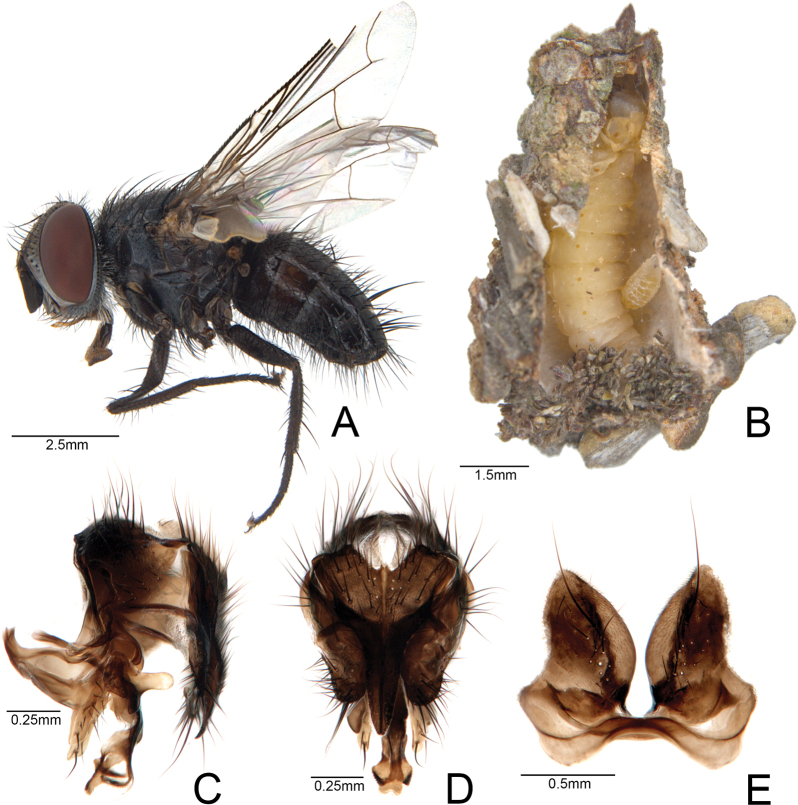
*Neophryxepsychidis*, a natural enemy of *Oiketicoidesgohadoensis*, sp. nov. **A** male of adult **B** parasitic view of larva **C** male genitalia of *N.psychidis*, lateral aspect **D** ditto, posterior aspect **E** ditto, 5^th^ sternite.

During the breeding of *O.gohadoensis* in this study, we discovered a parasitoid, *N.psychidis* (Fig. 4). This species is reported for the first time in Korea. According to the literature, this species is known to emerge from psychid cases and is distributed in China, Japan, and Russia (O’Hara et al. 2009). In the Palearctic region, 31 species of Tachinidae are known to be associated with at least 36 species of psychid moths, and at least seven species of psychid moths are known to be associated with *N.psychidis* (Tschorsnig 2017). DNA barcode sequences were generated (NCBI accession number PP983258). Multiple alignments using the BLAST tool in the NCBI database showed *N.psychidis* as the nearest neighbor (locality of reference data from Japan; 100%).

## Supplementary Material

XML Treatment for Oiketicoides

XML Treatment for Oiketicoides gohadoensis

## ﻿Additional information

### Author ORCIDs

Dong-June Lee https://orcid.org/0000-0003-1526-5127

Jongwon Kim https://orcid.org/0000-0002-1793-8041

Seung-Su Euo https://orcid.org/0009-0002-5061-8792

Jae-Seok Lee https://orcid.org/0000-0003-4502-7330

Hyeon Lee https://orcid.org/0009-0006-7467-7331

Seung Jin Roh https://orcid.org/0009-0002-6100-7604

### Data availability

All of the data that support the findings of this study are available in the main text.

## References

[B1] Arnscheid WR Sobczyk T (2023) Taxonomic review of the *Oiketicoides* species (Lepidoptera: Psychidae: Oiketicinae: Acanthopsychini) from Anatolia, the Middle East and Central Asia. Zootaxa 5239(3): 373–394. 10.11646/zootaxa.5239.3.3 37045093

[B2] Arnscheid WR Weidlich M (2017) Microlepidoptera of Europe. Vol. 8. Brill, Leiden, 423 pp.

[B3] Folmer O Black M Hoech W Lutz R Vrijenhoek R (1994) DNA primers for amplification of mitochondrial cytochrome c oxidase subunit I from diverse metazoan invertebrates. Molecular Marine Biology and Biotechnology 3(5): 294–299. 7881515

[B4] Heylaerts FJM (1881) Essai d’une monographie des psychides de la faune européene. Bulletin & Annales de la société entomologique de Belgique 25: 29–73.

[B5] Heylaerts F (1885) Psychides nouvelles ou moins connues de l’Empire Russe. Mémoires sur les Lépidoptères (Romanoff), St. Petersburg 2: 176–194 [pls 9, 10].

[B6] Jia QJ Wu CS (2023) Catalogue of the family Psychidae in China (Lepidoptera: Tineoidea). SHILAP Revista de Lepidopterología 51(203): 549–560. 10.57065/shilap.540

[B7] Kearse M Moir R Wilson A Stones-Havas S Cheung M Sturrock S Buxton S Cooper A Markowitz S Duran C Thierer T Ashton B Meintjes P Drummond A (2012) Geneious Basic: an integrated and extendable desktop software platform for the organization and analysis of sequence data. Bioinformatics 28(12): 1647–1649. 10.1093/bioinformatics/bts199 22543367 PMC3371832

[B8] Lederer J (1858) Noch einige syrische Schmetterlinge. Wiener entomologische Monatsschrift 2(5): 135–143.

[B9] Nieukerken EJ van Kaila L Kitching IJ Kristensen NP Lees DC Minet J Mitter C Mutanen M Regier JC Simonsen TJ Wahlberg N Yen S-H Zahiri R Adamski D Baixeras J Bartsch D Bengtsson BÅ Brown JW Bucheli SR Davis DR De Prins J De Prins W Epstein ME Gentili-Poole P Gielis C Hättenschwiler P Hausmann A Holloway JD Kallies A Karsholt O Kawahara AY Koster JC Kozlov M Lafontaine JD Lamas G Landry J-F Lee S Nuss M Park K-T Penz C Rota J Schintlmeister A Schmidt BC Sohn J-C Solis MA Tarmann GM Warren AD Weller S Yakovlev RV Zolotuhin VV Zwick A (2011) Order Lepidoptera Linnaeus, 1758. In: Zhang Z-Q (Ed.) Animal biodiversity: an outline of higher-level classification and survey of taxonomic richness.Zootaxa 3148: 212–221. 10.11646/zootaxa.3148.1.41

[B10] O’Hara JE Shima H Zhang C (2009) Annotated catalogue of the Tachinidae (Insecta: Diptera) of China. Zootaxa 2190: 1–236. 10.11646/zootaxa.2190.1.1

[B11] Regier J Mitter C Davis DR Harrison T Sohn J-C Cummings M Zwick A Mitter K (2015) A molecular phylogeny and revised classification for the oldest ditrysian moth lineages (Lepidoptera: Tineoidea), with implications for ancestral feeding habits of the mega-diverse Ditrysia. Systematic Entomology 40(2): 409–432. 10.1111/syen.12110

[B12] Saigusa T Sugimoto M (2014) Japanese species of the genus *Proutia* Tutt, 1899(Lepidoptera: Psychidae). Zootaxa 3869: 143–152. 25283906 10.11646/zootaxa.3869.2.3

[B13] Sobczyk T (2011) World Catalogue of Insects, Vol. 10 Psychidae (Lepidoptera). Apollo Books, Stenstrup, 467 pp. 10.1163/9789004261044

[B14] Tschorsnig HP. (2017) Preliminary host catalogue of Palaearctic Tachinidae (Diptera). http://www.nadsdiptera.org/Tach/WorldTachs/CatPalHosts/Cat_Pal_tach_hosts_Ver1.pdf [Accessed on: 2024-11-15]

[B15] Wehrli E (1928) Neue Psychiden und Geometriden (Lep.). Internationale Entomologische Zeitschrift 21(47): 454–457. 10.1002/mmnd.48119280102

